# *No-Reflow* nas Síndromes Coronarianas Agudas: Um Velho Inimigo ou uma Nova Fronteira?

**DOI:** 10.36660/abc.20210118

**Published:** 2021-05-06

**Affiliations:** 1 Hospital Israelita Albert Einstein São PauloSP Brasil Hospital Israelita Albert Einstein, São Paulo, SP - Brasil.; 2 Universidade de São Paulo Instituto do Coração São PauloSP Brasil Universidade de São Paulo Instituto do Coração, São Paulo, SP – Brasil.

**Keywords:** Isquemia Miocárdica, Doenças Cardiovasculares/mortalidade, Intervenção Coronária Percutânea, Infarto do Miocárdio, Doença da Artéria Coronária, Fatores de Risco, Resistência Vascular

Segundo a Organização Mundial da Saúde (OMS), a cardiopatia isquêmica é a principal causa de óbitos no mundo, tendo sido responsável por 16% dos óbitos no mundo em 2019.^1^ No entanto, devido à contínua evolução do tratamento médico e das técnicas de revascularização, tem-se observado um constante declínio nas taxas de mortalidade em síndromes coronarianas agudas (SCA) nos últimos anos.^2^

Atualmente, a intervenção coronariana percutânea (ICP) é o tratamento padrão-ouro para infarto do miocárdio com supradesnivelamento do segmento ST (IAMCST^3^ e uma opção terapêutica básica para SCA sem IAMCST^4^ e doença arterial coronariana estável.^5^ No entanto, e particularmente em pacientes com STEMI, a ICP pode ser muito desafiadora às vezes. Um dos eventos mais temidos durante a ICP no IAMCST é o fenômeno comumente denominado “*no-reflow*”, um comprometimento na perfusão miocárdica secundário à obstrução microvascular sem evidência angiográfica de obstrução coronariana. Descrito inicialmente em modelos animais^6^^,^^7^ também foi reconhecido em humanos nas décadas seguintes^8^^,^^9^ sendo descrito pela primeira vez após a ICP por IAMCST por Feld em 1992.^10^ Sua ocorrência está relacionada a desfechos de curto e de longo prazo após ICP,^11^^,^^12^ e está presente em mais de 20% dos pacientes submetidos a ICP primária por IAMCST.^13^

Em uma publicação recente,^14^ Rezkalla et al. revisaram exaustivamente o manejo do *no-reflow*, identificando muitos fatores de risco, como maior tempo de reperfusão, dilatação do balão de alta pressão, stents mais longos e também características clínicas do paciente, muitas das quais se sobrepõem às da doença arterial coronariana e SCA. Caso seja previsto o *no-reflow*, medidas técnicas e farmacológicas podem ser tomadas na tentativa de evitá-lo, possivelmente minimizando sua ocorrência e alertando o operador a agir prontamente caso ocorra.

De acordo com essa ideia, o artigo “Comparação entre Dois Escores de Risco quanto à Predição de Obstrução Microvascular Coronariana durante a Intervenção Percutânea Primária”,^15^ publicado na edição atual desta revista, explora a capacidade de dois escores de risco em prever a ocorrência de *no-reflow*. Ele compara o escore SAK, que utiliza parâmetros puramente clínicos (início dos sintomas até o tempo de insuflação do balão, nível de ACT na internação, classificação de Killip, idade, razão neutrófilos/linfócitos e níveis de glicose), com o escore ATI, cujos parâmetros são uma medida invasiva de resistência microvascular (MIRM) obtida por meio do escore de microcateter coronariano, idade e trombo na artéria culpada. Nesse estudo, ambos os escores tiveram bom desempenho, com o escore SAK apresentando uma AUC de 0,855. Nesse estudo, o *no-reflow* se mostrou mais comumente associado a pacientes com idade mais avançada, com maior tempo, maiores níveis de glicose, maiores níveis de creatinina sérica, maior contagem de leucócitos, classificação Killip III e aumento de biomarcadores de necrose miocárdica, o que está de acordo com a literatura médica atual. Porém, outros fatores, como hipertensão, dislipidemia, diabetes e tabagismo não estiveram relacionados à ocorrência do fenômeno, sugerindo que sua fisiopatologia ainda não esteja totalmente esclarecida. Além disso, não há dados sobre como o *no-reflow* foi tratado e se o tratamento resultou em melhora da resistência microvascular e possivelmente melhores resultados.

Em estudo publicado recentemente,^16^ Viana et al. compararam os escores SYNTAX e GRACE na predição de mortalidade cardiovascular e eventos coronarianos não fatais recorrentes após SCA. Ambos foram eficazes na predição de morte cardiovascular (estatística-C 0,80 vs. 0,89, p=0,19, para os escores SYNTAX e GRACE, respectivamente), mas o escore SYNTAX anatômico foi o único capaz de predizer eventos coronários recorrentes não fatais (estatística-C 0,64 vs. 0,50, p=0,027), sugerindo que complicações e resultados intraprocedimento, como *no-reflow*, não são levados em consideração ao usar escores de risco puramente clínicos para SCA.

Compreender toda a complexidade do ACS ainda parece estar fora do nosso alcance no momento. No entanto, perceber que o prognóstico e os resultados de tais pacientes resultam de diversos fatores clínicos e intraprocedimento pode ser o farol para nos ajudar a navegar por essas águas turbulentas e ainda não totalmente percorridas.

## References

[1] 1. World Health Organization.(WHO). World Health Statistics. Monitoring Health For The SDGs Sustainable Development Goals. Geneva;2020.

[2] 2. Fox KAA, Steg PG, Eagle KA, Goodman SG, Anderson FA, Granger CB, et al. Decline in rates of death and heart failure in acute coronary syndromes, 1999-2006. J Am Med Assoc. 2007;297(17):1892-900. 10.1001/jama.297.17.1892 17473299

[3] 3. Ibanez B, James S, Agewall S, Antunes MJ, Bucciarelli-Ducci C, Bueno H, et al. 2017 ESC Guidelines for the management of acute myocardial infarction in patients presenting with ST-segment elevation. Eur Heart J. 2018;39(2):119-77. 10.1093/eurheartj/ehx393 28886621

[4] 4. Collet J-P, Thiele H, Barbato E, Barthélémy O, Bauersachs J, et al. 2020 ESC Guidelines for the management of acute coronary syndromes in patients presenting without persistent ST-segment elevation. Eur Heart J. 2021;42(14):1289-367. 10.1093/eurheartj/ehaa575 32860058

[5] 5. Sousa-Uva M, Neumann FJ, Ahlsson A, Alfonso F, Banning AP, Benedetto U, et al. 2018 ESC/EACTS Guidelines on myocardial revascularization. Eur J Cardiothoracic Surg. 2019; 55(1):4-90. 10.1093/ejcts/ezy289 30165632

[6] 6. Kloner RA, Ganote CE, Jennings RB. The “no reflow” phenomenon after temporary coronary occlusion in the dog. J Clin Invest. 1974;54(6):1496-508. 10.1172/JCI107898 PMC301706 4140198

[7] 7. Krug A, Du Mesnil de Rochemont, Korb G. Blood supply of the myocardium after temporary coronary occlusion. Circ Res. 1966;19(1):57-62. 10.1161/01.res.19.1.57 5912914

[8] 8. Schofer J, Montz R, Mathey DG. Scintigraphic evidence of the “No reflow” phenomenon in human beings after coronary thrombolysis. J Am Coll Cardiol. 1985;5(3):593-8. 10.1016/s0735-1097(85)80381-8 3973255

[9] 9. Bates ER, Krell MJ, Dean EN, O’Neill WW, Vogel RA. Demonstration of the “no-reflow” phenomenon by digital coronary arteriography. Am J Cardiol. 1986;57(1):177-8. 10.1016/0002-9149(86)90976-8 3510523

[10] 10. Feld H, Lichstein E, Schachter J, Shani J. Early and late angiographic findings of the “no-reflow” phenomenon following direct angioplasty as primary treatment for acute myocardial infarction. Am Heart J. 1992;123(3):782-4. 10.1016/0002-8703(92)90520-6 1539531

[11] 11. Morishima I, Sone T, Okumura K, Tsuboi H, Kondo J, Mukawa H, et al. Angiographic no-reflow phenomenon as a predictor of adverse long-term outcome in patients treated with percutaneous transluminal coronary angioplasty for first acute myocardial infarction. J Am Coll Cardiol. 2000;36(4):1202-9. 10.1016/s0735-1097(00)00865-2 11028471

[12] 12. Harrison RW, Aggarwal A, Ou FS, Klein LW, Rumsfeld JS, Roe MT, et al. Incidence and outcomes of no-reflow phenomenon during percutaneous coronary intervention among patients with acute myocardial infarction. Am J Cardiol. 2013;111(2):178-84. 10.1016/j.amjcard.2012.09.015 23111142

[13] 13. Jaffe R, Charron T, Puley G, Dick A, Strauss BH. Microvascular obstruction and the no-reflow phenomenon after percutaneous coronary intervention. Circulation. 2008;117(24):3152-6. 10.1161/CIRCULATIONAHA.107.742312 18559715

[14] 14. Rezkalla SH, Stankowski R V., Hanna J, Kloner RA. Management of No-Reflow Phenomenon in the Catheterization Laboratory. JACC: Cardiovasc Intervent. 2017;10(3):215-23. 10.1016/j.jcin.2016.11.059 28183461

[15] 15. Xiao Y, Chen H, Liu D, Wang Y, Wang W, Zhang Q, et al. The Comparison between Two Risk Scores as for the Prediction of Coronary Microvascular Obstruction during Primary ercutaneous Intervention. Arq Bras Cardiol. 2021; 116(5):959-967. 10.36660/abc.20200115 PMC8121479 34008822

[16] 16. Viana MS, Correia VCA, Ferreira FM, Lacerda YF, Bagano GO, Fonseca LL, et al. Competência Prognóstica Distinta entre Modelo Clínico e Anatômico em Síndromes Coronarianas Agudas: Comparação por Tipo de Desfecho. Arq Bras Cardiol. 2020;115(2):219-25. 10.36660/abc.20190062 PMC8384280 32876188

