# Supplementary material for: No-Reflow nas Síndromes Coronarianas Agudas: Um Velho Inimigo ou uma Nova Fronteira?
Source: Arq Bras Cardiol. 2021 May 6;116(5):968–9. [Article in Portuguese] doi: 10.36660/abc.20210118 (PMC8121482; doi:10.36660/abc.20210118)
Supplement: Translation

# Translation: No Reflow in Acute Coronary Syndromes: An Old Foe or a New Frontier?

**Affiliations:** 1 São PauloSP Brazil Hospital Israelita Albert Einstein, São Paulo, SP - Brazil.; 2 São PauloSP Brazil Universidade de São Paulo Instituto do Coração, São Paulo, SP - Brazil.

**Keywords:** Myocardial Ischemic, Cardiovascular Diseases/mortality, Percutaneous Coronary Interventions, Myocardial Infarction, Coronary Artery Disease, Risk Factors, Vascular Resistance

According to the World Health Organization (WHO), ischemic heart disease is the leading cause of death worldwide, accounting for 16% of the world's deaths in 2019.^1^ However, due to continuous evolution in medical treatment and revascularization techniques, a steady decline in death rates in acute coronary syndromes (ACS) has been observed in recent years.^2^

Currently, percutaneous coronary intervention (PCI) is the gold-standard treatment for ST-elevation myocardial infarction (STEMI)^3^ and a mainstay therapeutic option for non-STEMI ACS^4^ and stable coronary artery disease.^5^ Nonetheless, and particularly in STEMI patients, PCI can be very challenging at times. One of the most dreaded events during PCI in STEMI is the phenomenon commonly referred as “no-reflow”, an impaired myocardial perfusion secondary to microvascular obstruction without angiographic evidence of coronary obstruction(6). Initially described in animal models,^6^^,^^7^ it was also recognized in humans in the following decades,^8^^,^^9^ being first described after PCI for STEMI by Feld in 1992.^10^ Its occurrence is related to poorer short- and long-term outcomes following PCI,^11^^,^^12^ and it is present in more than 20% of patients undergoing primary PCI for STEMI.^13^

In a recent publication,^14^ Rezkalla et al. thoroughly reviewed the management of no-reflow, identifying many risk factors, which include longer time to reperfusion, high-pressure balloon dilation, longer stents, and also clinical characteristics of the patient, many of which overlap with those of coronary artery disease and ACS. If no-reflow is anticipated, pharmacological and technical measures can be taken in an attempt to prevent it, potentially minimizing its occurrence and alerting the operator to promptly act in case it occurs.

According to this idea, the article “The Comparison between Two Risk Scores as for the Prediction of Coronary Microvascular Obstruction during Primary Percutaneous Intervention,”^15^ published in the current edition of this journal, explores the ability of two risk scores in predicting the occurrence of no-reflow. It compares the SAK score, which uses purely clinical parameters (symptom onset to balloon inflation time, ACT level on admission, Killip classification, age, neutrophil/lymphocyte ratio, and glucose levels), with the ATI score, whose parameters are an invasive measure of microvascular resistance (IMR) obtained via coronary microcatheter, age and thrombus score in the culprit artery. In this study, both scores performed well, with the SAK score presenting an AUC of 0.855. In this study, no-reflow was more commonly associated with older patients with longer reperfusion times, higher glucose levels, higher serum creatinine levels, higher leucocyte counts, Killip III classification and increased myocardial necrosis biomarkers, which is in accordance with current medical literature. However, other factors, such as hypertension, dyslipidemia, diabetes, and smoking were not related to the occurrence of the phenomenon, suggesting that its physiopathology is not yet fully understood. Also, there are no data regarding how no-reflow was treated and whether the treatment resulted in improvement of microvascular resistance and possibly better outcomes.

In a study recently published,^16^ Viana et al. compared the SYNTAX and GRACE scores in predicting cardiovascular mortality and recurring non-fatal coronary events after ACS. Both were effective in predicting cardiovascular death (C-statistic 0.80 vs. 0.89, p=0.19, for the SYNTAX and GRACE scores, respectively), but the anatomical SYNTAX score was the only one capable of predicting recurring non-fatal coronary events (C-statistic 0.64 vs. 0.50, p=0.027), suggesting that intra-procedural complications and outcomes, such as no-reflow, are not accounted for when using purely clinical ACS risk scores.

Understanding the full complexity of ACS still seems to be out of our reach at the moment. However, realizing that prognosis and outcomes of such patients result from numerous clinical and intra-procedural factors might be the beacon to help us navigate these troubled and not-yet-fully-charted waters.
